# Comparison of postoperative exercise capacity of patients who underwent sternal closure with SternaLock Blu and those with traditional sternal wire closure following cardiovascular surgery via sternotomy

**DOI:** 10.1007/s12055-023-01532-5

**Published:** 2023-07-17

**Authors:** Kiyoshi Tamura, Shogo Sakurai

**Affiliations:** Department of Cardiovascular Surgery, Soka Municipal Hospital, 2-21-1 Soka, Soka-shi, Saitama, 340-8560 Japan

**Keywords:** SternaLock Blu, Rigid plate fixation, Median sternotomy, Cardiovascular surgery, Exercise capacity

## Abstract

**Purpose:**

This study aimed to evaluate the efficacy of postoperative exercise capacity of SternaLock Blu (Zimmer Biomet, USA) for post-sternotomy patients who underwent cardiovascular surgery.

**Methods:**

We investigated 116 patients, including 35 women (mean age 70.3 ± 10.0 years), who underwent cardiovascular surgery by median sternotomy. Patients were candidate for using SternaLock Blu, such as obesity (body mass index  > 30/kg/m^2^), insulin-dependent diabetes mellitus, steroid administrating, undergoing coronary bypass artery grafting used bilateral internal thoracic artery. These patients were categorized into those with SternaLock Blu (S group, *n* = 47) or with sternal wires only (N group, *n* = 69) for closing sternotomy, and their data were analyzed.

**Results:**

Max Prince Henry Pain Scale (PHPS) was significantly low in the S group than in the N group (N vs. S = 2.7 ± 1.4 vs. 1.6 ± 1.7%, *p* < 0.001). Postoperative 6-min walk was significantly longer in the S group than in the N group (N vs. S = 346.2 ± 101.6 vs. 408.6 ± 104.6 m, *p* = 0.018). The length of intensive care unit (N vs. S = 4.9 ± 0.9 vs. 4.0 ± 1.2 days, *p* < 0.001) and hospitalization (N vs. S = 18.0 ± 5.0 vs. 15.2 ± 3.8 days, *p* = 0.006) were significantly shorter in the S group than in the N group.

**Conclusions:**

SternaLock Blu could keep exercise capacity, and might help reduce postoperative pain and medical treatment period for patients who underwent cardiovascular surgery.

## Introduction

Median sternotomy is a standard and general approach for cardiovascular surgery in the world. Though minimally invasive cardiac surgery is spread worldwide recently, median sternotomy is needed for many cardiovascular surgeries.

Though sternal wires are used for sternal fixation usually, other devices for rigid sternal fixation are used in cases. SternaLock Blu (Zimmer Biomet, USA) is one of devices for sternal fixation. Sternal reconstruction using SternaLock Blu improved bone healing and reduced early postoperative pain [1].

In Japan, SternaLock Blu is used for limited post-sternotomy patients who have one or multiple factors, such as obesity (body mass index; BMI  > 30/kg/m^2^), insulin administrating diabetes mellitus, steroid administrating, and undergoing coronary bypass artery grafting used bilateral internal thoracic artery (BITA). However, there are a few studies that report the usefulness of SternaLock Blu for those patients. This study aimed to evaluate comparison of postoperative exercise capacity of patients who underwent sternal closure with SternaLock Blu and those with traditional sternal wire closure following cardiovascular surgery via sternotomy.

## Subjects and methods

### Subjects

This study was approved by the Institutional Review Board of Soka Municipal Hospital, and written informed consent was obtained from all of the patients included in the study. This study was conducted in accordance with the ethical principles stated by the Declaration of Helsinki.

A total of 377 consecutive patients who underwent cardiovascular surgery via sternotomy at our hospital between March 2013 and June 2022. We included patients who were candidate for using SternaLock Blu, such as those with obesity (BMI  > 30/kg/m^2^), insulin-dependent diabetes mellitus (DM), steroid administrating, and undergoing coronary bypass artery grafting used BITA. And, we excluded patients who underwent emergency surgery and aortic surgery. Therefore, 116 patients (35 women, mean age 70.3 ± 10.0 years) were included. Heparin was infused in all patients for anticoagulation therapy (activated clotting time target:  > 350 s). Patients received blood transfusions (trigger  < 8 g/l hemoglobin).

### Materials and Methods

Between October 2013 to January 2017, SternaLock Blu was not used in cardiovascular surgery, and patients were referred to our hospital in February 2017. Basic sternal fixation is conventional wire fixation. This rigid plate fixation of SternaLock Blu is performed using one plate on the manubrium and two plates on the sternal body. Therefore, we categorized our study population into the N group (*n* = 69 patients who used sternal wires only for sternal reconstruction during the early study period) and the S group (*n* = 47 patients who used SternaLock Blu for sternal reconstruction during the latter study period). Perioperative data were collected, analyzed, and compared between the two groups. In the N group, sternal fixation is performed using two wires on the manubrium and four wires on the sternal body. The protocols of the cardiac surgery procedure were the same between the two time frames.

All patients were rehabilitated after cardiac surgery in our institution. The rehabilitation is started immediately after weaning from respirator. The first step of rehabilitation is sitting down on the bedside, and the final step is both a 500-m walk and stepping exercise of double floor-height. Patients rehabilitate this program step by step with the captive rehabilitator.

Preoperative 6-min walk is performed at the day before surgery, and postoperative 6-min walk is performed at the 10^th^ day after surgery with the captive rehabilitator. Both 6-min walk was stopped in the middle when those parameters were identified such as heart rate  > 130/min, blood pressure  > 180 mmHg, saturation of percutaneous oxygen  < 93%, and complaint of dyspnea or fatigue.

Frailty Score was defined by the revised Japanese version of the Cardiovascular Health Study criteria [2]. This Frailty Score is defined that 0 is no frail, 2–3 are pre-frail, and over 4 is frail.

Postoperative pain was evaluated by the Prince Henry Pain Scale (PHPS) [3]. PHPS Score is defined: 0 is no pain on cough, 1 is pain on cough but not on deep breaths, 2 is pain on deep breathing but not on rest, 3 is slight pain at rest, and 4 is severe pain at rest. Acetaminophen (1200 mg/day) is taken for analgesic drug in all patients. Patients with PHPS Score 4 were prescribed pentazocine. We invested max PHPS Score during hospitalization.

DM was defined as fasting blood glucose > 126 mg/dl, recent use of antidiabetic drugs, and/or serum hemoglobin A1c > 6.5%. Chronic kidney disease (CKD) was presented as an estimated glomerular filtration rate < 50 ml/min/1.73 m^2^. The diagnosis of surgical site infection (SSI) was based on the Centers for Disease Control and Prevention guideline [4, 5], as follows: (1) purulent discharge from the incision and (2) positive results of bacteria cultivation from the tissue or liquid obtained from the incision aseptically. Incisional SSI was defined as deep sternal infection. SSI was within 30 days of cardiovascular surgery.

The primary endpoint was the rate of SSI onset and the reduction of postoperative pain, and the secondary endpoint was the length of intensive care unit (ICU) and hospital stay.

Continuous data are expressed as mean ± standard deviation (SD) with ranges. Non-parametric data were analyzed using contingency tables; the Mann-Whitney *U* test was used. Parametric data were compared using the Student’s *t* test. The chi-squared test was used to analyze data presented in a contingency table. A *p* value  < 0.05 was considered statistically significant. EZR software (Easy R, version 2.0) was used for all statistical analyses [6].

## Results

This study included 116 patients (35 women, mean age 70.3 ± 10.0 years). No significant differences were found in preoperative characteristics such as age, sex, BMI, prevalence of hypertension, dyslipidemia, DM, CKD, smoking, peripheral arterial disease, serum hemoglobin levels, C-reactive protein, left ventricular ejection fraction, 6-min walk, and EuroSCORE II between two groups (Table 1). No significant differences were found in BMI  > 30 kg/m^2^, DM administrating insulin, and steroid administrating between the two groups, too.

**Table 1:** Preoperative characteristics

	N group (*n* = 69)	S group (*n* = 47)	*P* value
Age (year)	70.8 ± 10.4	69.3 ± 8.4	0.420
Sex (female)	21 (30.4%)	14 (29.8%)	0.941
BMI (kg/m^2^)	23.3 ± 3.6	24.3 ± 4.2	0.231
BMI > 30	12 (17.4%)	11 (23.4%)	0.430
Prevalence			
High blood pressure	60 (87.0%)	45 (95.7%)	0.115
Dyslipidemia	57 (82.6%)	40 (85.1%)	0.724
DM	53 (76.8%)	36 (76.6%)	0.979
DM administrating insulin	32 (46.4%)	15 (31.9%)	0.121
CKD	37 (53.6%)	22 (46.8%)	0.475
Smoking	10 (14.5%)	5 (10.6%)	0.548
PAD	10 (14.5%)	7 (14.9%)	0.953
Steroid administrating	6 (8.7%)	2 (4.2%)	0.247
Hb (g/dl)	12.4 ± 1.8	13.0 ± 2.8	0.165
CRP (mg/dl)	0.5 ± 0.9	0.3 ± 0.5	0.209
EF (%)	59.5 ± 13.1	55.6 ± 15.8	0.179
6-min walk (m)	373.6 ± 100.0	370.8 ± 132.3	0.915
EuroSCORE II (%)	2.9 ± 2.6	3.1 ± 3.3	0.818
Frailty Score	1.7 ± 0.9	1.4 ± 0.9	0.129

*BMI*, body mass index; *DM*, diabetes mellitus; *CKD*, chronic kidney disease; *PAD*, peripheral artery disease; *Hb*, hemoglobin; *CRP*, C-reactive protein; *EF*, ejection fraction

Though no statistically significant intergroup differences were observed in the rate of operation types, coronary artery bypass grafting (CABG) used BITA was significantly more in the S group compared with the N group (Table 2).

**Table 2:** Operative characteristics

	N group (*n* = 69)	S group (*n* = 47)	*P* value
CABG	43 (62.3%)	35 (74.5%)	0.174
Valve surgery	26 (37.7%)	12 (25.5%)	0.174
CABG used BITA	0 (0.0%)	12 (25.5%)	< 0.001
Operative time (min)	394.2 ± 132.4	394.6 ± 94.5	0.996
Cardiopulmonary bypass time (min)	224.6 ± 97.3	192.4 ± 79.2	0.167
Use of IABP	2 (2.9%)	2 (4.3%)	0.697
Blood transfusion	51 (73.9%)	30 (63.8%)	0.249

*CABG*, coronary artery bypass grafting; *BITA*, bilateral internal thoracic artery, *IABP*, intra-aortic balloon pumping

Though no significant difference was found in SSI (N vs. S = 4.3 vs. 0.0%, *p* = 0.147), PHPS was significantly low in the S group than in the N group (N vs. S = 2.7 ± 1.4 vs. 1.6 ± 1.7%, *p* < 0.001) (Table 3). The duration needed for rehabilitation completion (N vs. S = 10.2 ± 1.8 vs. 9.1 ± 1.9 days, *p* = 0.026) was significantly shorter in the S group than in the N group, and postoperative 6-min walk was significantly longer in the S group than in the N group (N vs. S = 346.2 ± 101.6 vs. 408.6 ± 104.6 m, *p* = 0.018). The length of ICU (N vs. S = 4.9 ± 0.9 vs. 4.0 ± 1.2 days, *p* < 0.001) and hospitalization (N vs. S = 18.0 ± 5.0 vs. 15.2 ± 3.8 days, *p* = 0.006) were significantly shorter in the S group than in the N group.

**Table 3:** Postoperative characteristics

	N group (*n* = 69)	S group (*n* = 47)	*P* value
Surgical site infection	3 (4.3%)	0 (0.0%)	0.147
Re-sternotomy	3 (4.3%)	0 (0.0%)	0.147
Re-intubation	2 (2.9%)	0 (0.0%)	0.239
Atrial fibrillation	27 (39.1%)	13 (27.7%)	0.186
Max Prince Henry Pain Scale	2.7 ± 1.4	1.6 ± 1.7	< 0.001
6-min walk (m)	346.2 ± 101.6	408.6 ± 104.6	0.004
Duration needed for rehabilitation completion (day)	10.2 ± 1.8	9.1 ± 1.9	0.026
ICU stay (day)	4.9 ± 0.9	4.0 ± 1.2	< 0.001
Hospitalization (day)	18.0 ± 5.0	15.2 ± 3.8	0.006
Hospital death	3 (4.3%)	1 (2.1%)	0.565

*ICU*, intensive care unit

## Discussions

In this study, no significant difference was found in the incidence of SSI, postoperative pain was significantly reduced in the S group than in the N group, and ICU and hospital stay was significantly shorter in the S group than in the N group, too.

In cardiovascular surgery, SSI was reported 1–9% of patients [7–9]. In this study, the incidence rate of SSI was usual (in total 7.8%). To prevent SSI, we followed the Centers for Disease Control and Prevention (CDC) guideline [10]. The factors to the development of SSI have been reported to patients with high BMI, history of alcoholism, chronic heart disease, and DM [11, 12]. These are mainly because those factors cause a widespread decrease in the immune function thereby causing delayed wound healing. However, Song et al. reported that there was a significant decrease in the incidence of postoperative mediastinitis in patients who benefited from sternal closure with rigid plate fixation compared with patients whose sternum was closed with a wire [13]. In this study, no significant differences were found in BMI  > 30 kg/m^2^, DM administrating insulin, and steroid administrating between the two groups, and no significant difference was found in the incidence of SSI between the two groups too (Tables 1, 2, and 3). SternaLock Blu might not increase SSI. Patients who benefited from sternal closure with rigid plate fixation showed a significant decrease in the incidence of postoperative mediastinitis when compared to similar population of patients whose sternum was closed with a wire. Because this study has small sample size, we could not show the reduction of SSI in this report. Further prospective studies that include a large number of patients are warranted to gain a deeper understanding of this subject.

In this study, PHPS was significantly low in the S group compared with the N group (Table 3). Raman et al. reported that SternaLock Blu reduced early postoperative pain [1]. Billè et al. confirmed that external fixation of a bone fracture relieved pain and improved quality of life [14]. Sakashita et al. reported that the fixation of sternotomy with Super FIXSORB MX40 promoted bone stability and decreased post-surgical pain [15]. The external fixation for sternotomy using SternaLock Blu might increase bone stability and reduce postoperative pain.

In Table 3, the duration needed for rehabilitation completion was significantly shorter in the S group than in the N group. All patients who underwent cardiovascular surgery in our institution performed 6-min walk in pre- and postoperative term. Though no significant difference was found in preoperative 6-min walk between the two groups, postoperative 6-min walk was significantly longer in the S group compared with the N group (Tables 1 and 3). The 6-min walk test was reported for a measure of exercise capacity in patients with chronic heart failure [16]. Stable stabilization with rigid plate fixation reduces postoperative pain, and the stabilization might cause short rehabilitation and keep for exercise capacity of patients.

The length of ICU and hospitalization were significantly shorter in the S group than in the N group (Table 3). Snyder et al. reported that postoperative length of stay was significantly shorter in the plate group compared with the wire group [17]. They presented that the reduction of hospital stay might be attributable to the improved postoperative comfort, pulmonary toilet, and mobility provided by a more stable sternal closure. Additionally, Raman et al. reported that sternal healing was superior in rigid plate fixation patients at both 3 and 6 months compared with wire cerclage-only group and that pain scores and narcotic usage were lower in rigid plate fixation patients compared with wire cerclage-only group [1]. In this study, postoperative 6-min walk was significantly longer in the S group compared with the N group (Table 3). The excellent exercise capacity preserved by rigid plate fixation might reduce the length of ICU and hospital stay.

This study is mainly limited by its retrospective, small-scale, and single-center study design. In addition, only 116 patients who underwent cardiovascular surgery were included. The duration of SternaLock Blu use (N or S group) was different. The protocols of cardiac surgery procedure were the same between the two time frames. Additionally, we analyzed the length of ICU and hospitalization in patients excluded this study (*n* = 261). We categorized those patients into the early phase group (*n* = 90 patients who underwent cardiovascular surgery from March 2013 to January 2017) and the late phase group (*n* = 171 patients who underwent cardiovascular surgery from February 2017 to June 2022). No significant differences were found in preoperative and operative characteristics (data not shown), and no significant differences were found in the length of ICU (early phase vs. late phase = 6.9 ± 6.8 vs. 6.1 ± 11.6 days, *p* = 0.483) and hospitalization (early phase vs. late phase = 26.7 ± 23.9 vs. 24.5 ± 22.8 days, *p* = 0.429). So, we thought that the phase did not affect the length of ICU and hospitalization in this study.

Thus, prospective studies with a large number of patients are needed to gain a deeper understanding of this subject.

## Conclusions

SternaLock Blu could keep exercise capacity and might help reduce postoperative pain and medical treatment period for patients with median sternotomy. SternaLock Blu might be safe and useful for patients who underwent cardiovascular surgery by median sternotomy.

## Data Availability

If required, it is possible.

## Code availability

EZR version 2.0 software program was used for all calculations.
